# Internet memes related to the COVID-19 pandemic as a potential coping mechanism for anxiety

**DOI:** 10.1038/s41598-021-00857-8

**Published:** 2021-11-12

**Authors:** Umair Akram, Kamila Irvine, Sarah F. Allen, Jodie C. Stevenson, Jason G. Ellis, Jennifer Drabble

**Affiliations:** 1 MEMElab, Department of Psychology, Sociology and Politics, Sheffield Hallam University, Collegiate Crescent, Sheffield, South Yorkshire S10 2BP UK; 2 School of Psychology, University of Lincoln, Lincoln, UK; 3 School of Social Sciences, Humanities and Law, Teesside University, Middlesbrough, UK; 4 Department of Psychology, Faculty of Health & Life Sciences, Northumbria University, Newcastle upon Tyne, UK

**Keywords:** Human behaviour, Anxiety

## Abstract

This study examined whether significantly anxious individuals differed from non-anxious individuals in their perceptual ratings of internet memes related to the Covid-19 pandemic, whilst considering the mediating role of emotion regulation. Eighty individuals presenting clinically significant anxiety symptoms (indicating ≥ 15 on the GAD-7) and 80 non-anxious controls (indicating ≤ 4) rated the emotional valance, humour, relatability, shareability, and offensiveness of 45 Covid-19 internet memes. A measure of emotion regulation difficulties was also completed. The perception of humour, relatability, and shareability were all greater amongst anxious individuals relative to non-anxious controls. These differences were not mediated by emotion regulation deficits. Internet memes related to the current Covid-19 pandemic may tentatively serve as coping mechanism for individuals experiencing severe symptoms of anxiety.

## Introduction

The recent outbreak of a novel respiratory coronavirus disease (termed Covid-19 as from here on) caused by severe acute respiratory syndrome coronavirus 2 (SARS-CoV-2) has become a global pandemic. Whilst most individuals with Covid-19 display mild to moderate symptoms, almost 15% present severe pneumonia and approximately 5% progress to multiple organ failure, hypoxia and acute respiratory distress syndrome^1,2^. To prevent the spread of Covid-19, governments worldwide typically enforce a number of health-related measures (i.e., promoting social distancing and face masks, quarantining, applying curfews, and social lockdowns), which may infringe personal freedoms and create financial uncertainty^1^. Certainly, these factors combined with the prolonged experience of being in a pandemic, may disturb cognitive (i.e., cognitive reappraisal) and behavioural (i.e., expressive suppression) processes that maintain stable functioning of the emotion regulation system^13,14^. Consequently, the onset of psychiatric difficulties may be promoted in those with no history of such difficulties, whilst also serving to accentuate the severity of pre-existing difficulties^2,3^. Indeed, recent work has observed an increase in the population prevalence of common mental health difficulties in a number of countries^4–7^. For example, a twofold increase in the prevalence of anxiety (20% in April 2020 vs. 11% in 2018) and increase of depressive symptoms (16% in April 2020 vs. 10% in 2018) was reported by Sciensano^6^. Likewise, Huang and Zhao^4^ determined an increased prevalence of generalised anxiety, symptoms of depression, and sleep disturbances in the Chinese general population. Similar increases in mental health difficulties have been evidenced in Columbia^5^, Iran^7^, Ireland^8^, the United States^9^ and United Kingdom^10^.

Not all people are affected by emotional distress caused by traumatic incidents such as the current Covid-19 pandemic^11^. In the context of Covid-19, the extent of trait resilience and ability to deploy adequate emotion regulation strategies appear to be related to a greater ability to cope with emerging trauma and psychiatric symptoms^12–14^. Whereas experience of fear may be considered an adaptive emotion that serves to mobilize energy to deal with potential threat^15^. For example, by activating appropriate safety behaviours (e.g., social distancing, hand sanitising) which may reduce contamination. However, these behaviours are known to also increase the experience of fear and anxiety related to health and contamination^15–18^. More recent work provides suggestive support for a number of basic psychosocial coping behaviours which may attenuate Covid-19 anxiety^3^. Specifically, in a large sample of the Spanish general population, Fullana and colleagues^3^ found reduced anxiety to be associated with a healthy and balanced diet, and avoiding news related to the current pandemic. Cowan^19^ examined the concerns of people with lived experiences of mental health and their supporters, health or social care practitioners and researchers, and the UK general public in March 2020, when the country entered a formal lockdown. In each of the subpopulations, anxiety emerged to be the most prominent concern. This included accentuation of pre-existing anxiety, first onset generalized anxiety, existential anxiety, and health-related anxiety. Here, individuals were particularly worried about loved ones and one’s own health in the context of the pandemic, in addition to worries about population compliance (or disregard) with government guidelines. Moreover, the outcomes also demonstrated that increased consumption of media and social media were reported to fuel the experience of anxiety^19^. Preliminary research highlights the role of emotion dysregulation in predicting early signs of distress and anxiety related to Covid-19^14^. Using a prospective design, Tyra and colleagues^14^ found an increased presentation of emotion regulation deficits predicted future onset of acute stress in response to the Covid-19 pandemic. Interestingly, this effect was largely driven emotional nonacceptance and the perception of limited access to emotion regulation strategies. These outcomes are in line with cross-sectional observations demonstrating significant relationships between Covid-19 related anxiety and emotion regulation difficulties^13,14^.

The experience of humour is considered vital in maintaining physical and psychological wellbeing^20^. Indeed, the use of humour is consistently related to wellbeing^20,21^, serves to regulate emotions^22–24^ and is considered an effective coping mechanism in the face of negative and stressful life events^24–27^. In healthy subjects, humour affiliative and self-enhancing humour appears to effectively down-regulate negative and up-regulate positive emotions^28^. To that end, the use of humour has been examined as a potential coping mechanism in relation to the current pandemic^26–32^. In the Italian general population, Bischetti and colleagues^31^ found humour related to the Covid-19 pandemic was generally perceived as aversive. However, those who reported using humour as an emotional coping mechanism in general were open to engaging with humour related to the pandemic^28^. Qualitative research following the Covid-19 pandemic found that positive emotion, a desire for cohesion, and desire for closeness to be prominent motivational factors when sharing what was considered as amusing content on social media^26^.

Expanding on perceived humour, the role of internet meme use has also been explored in relation to the current pandemic^27,32^. This line of equerry appears worthwhile considering improved mental health outcomes associated with greater use of social media activity directly related to the current pandemic^33–35^. Typically, internet memes depict humorous social commentaries that are contextually relevant to a particular demographic of individuals^36^. They have become a vital aspect of digital culture which are largely well established in the media by consistently maintaining current references that often step into cultural and political domains. Dynel^30^ performed discourse analysis on a set of Covid-19 memes specifically related to mask wearing which were shared on social media webpages. The content of these memes was predominately related to use of peculiar household items to create homemade masks; and beliefs surrounding the efficacy of mask use. Here, memes based on images of public places were largely focused on extreme or peculiar masks and outfits that were presumably used in a preventative way, whereas self-taken images often depicted humour orientated mask parodies. Finally, many of the memes were shared virally, with the initial images being used to create various iterations^27^. Hussein and Aljamili^32^ examined the perceived humour and mood improving perception of memes and caricatures related to the Covid-19 pandemic amongst a sample of the Jordanian population. Whilst no formal comprehensive analysis was performed, demographic results indicated that the majority of the sample perceived the presented memes and caricatures as being humorous with the potential to improve mood^32^.

Evidence also suggests that internet memes related to psychiatric difficulties (i.e., anxiety and depression) may be beneficial for populations who experience such difficulties^37–40^. In a survey of 133 college students, 47% of individuals reported engaging with memes as a way of alleviating psychiatric symptoms^40^. More recent research found perceptual differences between subclinical depressed and non-depressed individuals in their interpretation of depressive memes^38,41^. Here, when compared with non-depressed controls, depressed individuals reported increased (i) perceptual ratings of humour, (ii) relatability, (iii) shareability, and (iii) mood improving potential of depressive memes. These differences were mediated by deficits in the ability to deploy adaptive emotion regulation strategies. This work tentatively suggests that depressive memes promote: a humorous take on negative experiences; and perceived support by connecting with others experiencing related symptoms^34^.

Considering the relationship between social media consumption and increased population prevalence of generalised anxiety^17^, this exploratory study sought to determine how internet memes related to the Covid-19 pandemic may be differentially perceived, and perhaps in a more positive manner, by individuals who are experiencing severe levels of anxiety relative to their non-anxious counterparts. More specifically, we examined group differences (i.e., anxious vs. non anxious controls) in the perception of emotional valance, humour, relatability, shareability, and offensiveness of internet memes related to the COVID-19 pandemic. As humour may facilitate cognitive reappraisal and improve emotion regulation difficulties^28^ typically observed amongst individuals exhibiting symptoms of and/or a disorder of anxiety^42–45^, we additionally examined the extent to which deficits in emotion regulation mediated any confirmed perceptual differences.

## Method

### Participants

In accordance with the British Psychological Society's Code of Human Research Ethics and the host institution's Research Ethics Policy, the study was approved by the Sheffield Hallam University University's Research Ethics Committee [protocol number: ER28392099], and all participants provided online informed consent prior to data collection. A cross-sectional online questionnaire-based survey was implemented comprising of questions designed to examine emotion dysregulation, symptoms of anxiety and depression, social media and meme use, and the perception of internet memes related to the pandemic. The survey was advertised to members of the general population through social media (i.e., Facebook, Twitter) and online internet forums (i.e., Reddit), and students from four institutional course participation schemes. This resulted in a sample of N = 513 individuals who either began or clicked on a hyperlink to the survey which was delivered using the Qualtrics platform (Qualtrics, Provo, UT). Only complete cases were used in the analysis due to the ethical right to withdraw from the survey at any time. The data was also examined for duplicate responses based on matching IP addresses, where none were found. Therefore, N = 410 respondents (mean age = 22.65 ± 7.46 years, range 18–60, 84% female) providing complete data (final response rate = 80%) for the variables of interest were entered into the final analysis.

### Materials

#### Anxiety

The 7-item Generalized Anxiety Disorder Scale (GAD-7)^46^ is a validated practical self-report anxiety questionnaire used in primary care. The tool asks respondents how often, during the last 2 weeks, they have been bothered by each of the seven core symptoms of generalized anxiety disorder (e.g., “not able to stop or control worrying”). Response choices are 0 = not at all; 1 = several days; 2 = more than half the days; and 3 = nearly every day. Total scores range between 0 and 21, with categorical cut offs of: 0–4 indicating minimal anxiety; 5–9 indicating mild anxiety; 10–14 indicating moderate anxiety; and 15–21 indicating severe anxiety levels. The GAD-7 has been shown to exhibit good reliability, as well as criterion, construct, factorial, and procedural validity^46^. The Cronbach’s alpha in the current study was *α* = 0.97.

#### Emotion regulation

The Emotion Regulation Questionnaire (ERQ)^47^ assessed emotion regulation strategies using two independent subscales: cognitive reappraisal (6 items, e.g., “When I want to feel less negative emotion, I change the way I'm thinking about the situation”) and expressive suppression (4 items, e.g., “I keep my emotions to myself”). Here, cognitive reappraisal refers to active alteration of how one thinks about potentially emotion-eliciting events, whereas expressive suppression involves behavioural changes in response to such events. For each subscale, respondents were asked to rate items using a 7-point Likert scale ranging from 1 (Strongly disagree) to 7 (Strongly agree). Total scores for cognitive reappraisal range between 7 and 28, whereas total scores for expressive suppression range between 7 and 42. Higher scores on each subscale indicate increased use of each strategy. The authors specify that each subscale should remain independent, with no alteration to wording or inception of subscales to create a composite score. This measure has been shown to have good internal consistency with Cronbach's α values greater than 0.80 for both subscales^48^. The Cronbach's alpha of the reappraisal and suppression subscales for the present sample were *α* = 0.91 and 0.80, respectively.

#### Covid proximity

To control for Covid proximity, participants were asked to indicate whether they have had Covid-19, a relative or close friend had Covid-19, they lost a relative or close friend to Covid-19. Where at least one of these scenarios was indicated as being true, participants were considered to be in high proximity, whereas those indicating a null response to all three questions were considered to be in low proximity. In the case that group differences emerged in the extent of Covid proximity, this was controlled for in the analysis.

### Pictorial stimuli

**Figure 1:**
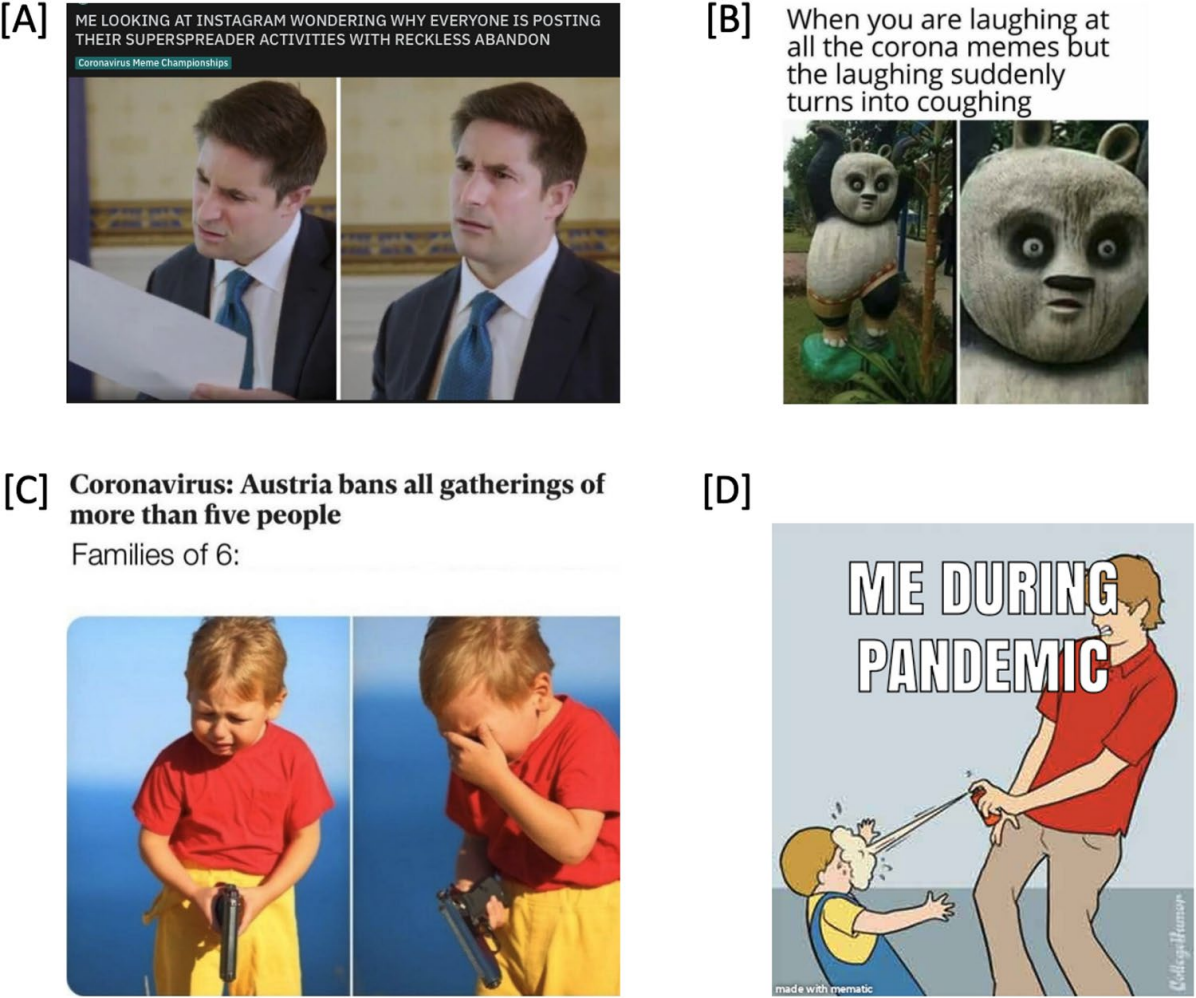
Four examples (**A**–**D**) of COVID-19 internet memes. All images used were gathered from the public domain each marked with either the Public Domain Mark 1.0 or CC0 1.0 Universal licence. No alterations were made. Title and Artist: Unknown. Image Source: Snappygoat.

In the absence of an existing picture set comprised of memes relating to the Covid-19 pandemic, a new set was developed and validated within the context of this study. Pictorial memes relating to the Covid-19 pandemic were obtained from an online forum, Reddit, which hosts a number of subreddits dedicated sharing internet memes specifically related to Covid-19 (i.e., /Coronavirusmemes), some which even hold ‘Coronavirus Meme Championships’. Specifically, we selected 45 of the highest rated (i.e., most upvoted) memes directly related to the pandemic. Each was comprised of an image paired with a short amount of text (see s1 for images). The final stimuli set included in the survey was agreed by all members of the research team and were standardised for presentation size 800 × 800 px (see Fig. 1 for examples).

### Procedure

Participants completed the online questionnaire, in which they were presented the series of 45 pictorial memes in randomized order. Using a 5-point Likert scale ranging from strongly disagree (= 1) to agree (= 5), participants reported the extent to which each meme was considered to be: negative; personally relatable; funny; offensive; something they would share with other people; and related to the COVID-19 pandemic. Following the pictorial meme ratings, the GAD-7, ERQ and in-house questions examining Covid proximity were administered.

### Statistical analyses

#### Stimuli validation

Jamovi (The Jamovi Project, 2020) was used to conduct statistical analyses of the data. Preliminary analysis confirmed the extent each pictorial meme was indeed related to the Covid-19 pandemic, with those deemed unrepresentative consequently discarded from final analysis. Specifically, based on mean ratings which failed to exceed ≥ 4 when asked to indicate the extent to which each meme was related to the Covid-19 Pandemic (strongly disagree = 1 to strongly agree = 5), N = 9 memes were discarded. Paired samples t-tests confirmed that the mean relatability ratings of the discarded memes (3.80 ± 0.81) were significantly lower than those which were retained (4.28 ± 0.60) for analysis (*t*(409) = 22.11), *p* < 0.0001). Examination of internal consistency (Cronbach’s α) for the final set of pandemic memes yielded a high degree of consistency: α = 0.94. Therefore, this process left a set of N = 36 memes that were related to the COVID-19 pandemic with a degree of confidence.

#### Participant grouping

Participants were first grouped based on the severity of reported anxiety symptoms. Using the GAD-7, individuals with a score of: ≤ 4 (Mean = 2.02 ± 1.37; Range = 0–4) were placed into the control group (N = 100: mean age = 23.88 ± 9.61; 81% female); and ≥ 15 (Mean = 18.23 ± 2.07; Range = 15–21) into the severe anxiety group (N = 80: mean age = 21.69 ± 5.02; 89% female). Data from N = 230 individuals who did not meet the criteria for either group were discarded at this point. Following, mean perception ratings for the final set of N = 36 memes were calculated. This was conducted for each parameter assessed. Specifically: (a) valance, as assessed by asking the extent to which each meme was considered to be negative; (b) personally relatable (i.e., relatable); (c) humor (or funniness); (d) offensive; (e) something they would share with other people (i.e., shareable); and related to the Covid-19 pandemic.

#### Analysis

A 2 (Group) × 5 (Rating Type) mixed model analysis of variance (MANOVA) was employed with anxiety groupings as independent variable and rating scores as the dependant variable. This was followed by a series of hierarchical linear regression analyses (using the enter method) to determine the extent to which group status (control vs. anxiety) and emotion regulation difficulties influence the perception of internet memes related to Covid-19. For example, group status (step 1), and emotional regulation subscales (cognitive reappraisal and expressive suppression: step 2) were entered as predictor variables, with rating type (e.g., humor) as the dependent variable. Finally, regression-based multiple mediation modelling was used with the MEDMOD plugin for Jamovi (The Jamovi Project), examined direct and indirect associations between group status and rating scores, via any significant covariates. Significance was considered at the p < 0.05 level.

## Results

Mean scores on the GAD-7 and ERQ, as well as meme ratings for each group, are displayed in Table 1. Groups did not differ in age (*t*(178) = 1.85, *p* > 0.05), sex (*X*(1) = 2.03, *p* > 0.05) or Covid proximity (*X*(1) = 0.01, *p* > 0.05). The Wilk's Lambda multivariate test of overall differences amongst groups was significant (*F*(1,78) = 2.69, *p* = 0.023). Univariate between-subjects tests demonstrated that humour (*F*(1,78) = 2.83, *p* = 0.021), relatability (*F*(1,78) = 6.16, *p* = 0.014), and shareability (*F*(1,78) = 13.52, *p* = 0.001) ratings differed significantly between the control and anxiety groups. More specifically, the anxiety group rated the memes as being significantly more humorous (3.67 ± 0.69), relatable (2.71 ± 0.74) and sharable (2.75 ± 0.97) when compared to controls (humour: 3.42 ± 0.75; relatable: 2.43 ± 0.77; sharable: 2.25 ± 0.83). No group differences in perceived valance and ratings of offensiveness were observed (see Table 1 for all group differences and effect sizes).

**Table 1:** Ratings of memes for the control and severe anxiety symptoms groups whilst observing COVID-19 memes (means ± standard deviation).

	Anxiety group	Control group	F	P	Cohen’s *d*
**Meme ratings**					
Valance	1.81 ± 0.73	1.78 ± 0.78	0.09	0.770	0.04
Humour	3.67 ± 0.69	3.42 ± 0.75	5.42	0.021*	0.35
Relatable	2.71 ± 0.74	2.43 ± 0.77	6.12	0.014*	0.37
Shareable	2.75 ± 0.97	2.25 ± 0.83	13.52	0.001***	0.66
Offensive	1.63 ± 0.65	1.60 ± 0.65	0.07	0.799	0.05
Anxiety	18.23 ± 2.07	2.02 ± 1.37	3950.25	0.001***	9.24
ER: cognitive reappraisal	23.28 ± 9.20	28.54 ± 5.92	21.58	0.001***	0.68
ER: expressive suppression	15.98 ± 5.71	13.84 ± 5.26	6.78	0.010**	0.34

*Sig at < 0.05, ** < 0.01, *** < 0.001.

A bootstrapped (1000 resamples) linear regression analysis demonstrated that group status significantly predicted humour ratings (step 1, *t*(178) = 2.33, *p* = 0.021), such that individuals experience anxiety expressed greater humour ratings. After accounting for both subscales of emotion regulation in the second step, group status (*t*(176) = 2.92, *p* = 0.004) and cognitive reappraisal (*t(*176) = 2.03, *p* = 0.044) but not expressive suppression (*t*(178) =  − 0.50, *p* = 0.617) significantly predicted ratings of humour (see Table 2a). However, whilst group status significantly predicted relatability (step 1, *t(*178) = 2.48, *p* = 0.014) and shareability (step 1, *t*(178) = 3.66, *p* = 0.001), cognitive reappraisal (relatability: *t*(176) = 1.93, *p* = 0.055; shareability: *t*(176) = 1.09, *p* = 0.276) and expressive suppression (relatability: *t*(176) =  − 1.25, *p* = 0.215; shareability: *t*(176) = − 0.37, *p* = 0.709) failed to predict the extent of ratings in the subsequent steps (see Table 2b,c respectively).

**Table 2:** Linear regression analyses with rating type as the dependant variables; and group status and emotion regulation subscales as predictors.

Predictors	df	R^2^	β	*t*	Sig.
**[A] Humour**					
Step 1	178	0.030			
Group status			0.25	2.33	0.021*
Step 2	176	0.053			
Group status			0.34	2.92	0.004**
Cognitive reappraisal			0.01	2.03	0.044*
Expressive suppression			− 0.01	− 0.50	0.617
**[B] Relatable**					
Step 1	178	0.033			
Group status			0.28	2.48	0.014*
Step 2	176	0.061			
Group status			0.38	3.17	0.002**
Cognitive reappraisal			0.01	1.93	0.055
Expressive suppression			− 0.01	− 1.25	0.215
**[C] Sharable**					
Step 1	178	0.071			
Group status			0.49	3.66	0.001***
Step 2	176	0.078			
Group status			0.56	3.83	0.001***
Cognitive reappraisal			0.01	1.09	0.276
Expressive suppression			− 0.01	− 0.37	0.709

1000 bias corrected bootstrapped samples. ERQ, Emotion Regulation Questionnaire.

*Sig at < 0.05, ** < 0.01, *** <0.001.

### Mediating role of emotion regulation

Based on the outcomes of the regression analyses, the mediating effects of cognitive reappraisal and expressive suppression were further examined using the MEDMOD plugin for Jamovi. Bootstrapping with 1000 bias-corrected and accelerate resamples and 95% confidence intervals were used, and the Sobel test (z) was used to indicate the hypothesized mediation effects. As demonstrated in Table 3, the results demonstrated significant direct effects of group status and ratings of humour (*z* = 3.13, *p* = 0.002), relatability (*z* = 3.33, *p* = 0.001) and shareability (*z* = 3.80, *p* = 0.001). Whilst no significant indirect effects of emotion regulation were observed between group status and each meme rating type, the effect of cognitive reappraisal on perceived humour approached statistical significance (*z* = − 1.90, *p* = 0.057).

**Table 3:** Examination of the mediating effect of emotion regulation, with meme ratings as the dependent variables and group status as the predictor.

	Estimate	SE	95% CI estimate		β	Z	Sig.
			Lower	Upper			
**[A] Humour**							
Group ⇒ CR ⇒ humour	− 0.08	0.04	− 0.17	− 0.01	− 0.05	− 1.90	0.057
Group ⇒ ES ⇒ humour	− 0.01	0.02	− 0.07	0.02	− 0.01	− 0.47	0.636
Group ⇒ humour	0.34	0.11	0.12	0.55	0.23	3.13	0.002**
**[B] Relatable**							
Group ⇒ CR ⇒ relatable	− 0.08	0.04	− 0.18	− 0.01	− 0.05	− 1.84	0.065
Group ⇒ ES ⇒ relatable	− 0.03	0.02	− 0.10	0.00	− 0.02	− 1.15	0.252
Group ⇒ relatable	0.38	0.12	0.15	0.59	0.25	3.33	0.001***
**[C] Sharable**							
Group ⇒ CR ⇒ sharable	− 0.05	0.05	− 0.17	0.04	− 0.03	− 0.95	0.343
Group ⇒ ES ⇒ sharable	− 0.01	0.03	− 0.09	0.03	− 0.01	− 0.34	0.734
Group ⇒ sharable	0.55	0.15	0.25	0.83	0.29	3.80	0.001***

Mediation model, 1000 bias corrected bootstrapped samples; CR, cognitive reappraisal; ES, expressive suppression.

** < 0.01, *** < 0.001.

## Discussion

This study sought to determine how internet memes related to the Covid-19 pandemic may be differentially perceived by individuals experiencing severe levels of anxiety when compared with their non-anxious counterparts and whether this was influenced by emotional regulation. With the exception of valance and offensiveness ratings, groups differed in their interpretation of memes related to Covid-19. More specifically, the perception of humour, relatability and shareability were all greater amongst individuals with symptoms of anxiety relative to non-anxious controls. Whilst linear regression analysis suggested that these perceptual differences may be mediated by deficits in emotion regulation, mediation modelling failed to support this notion.

Many of the presently used internet memes related to Covid-19 pandemic may be considered negative in nature, depicting themes of isolation and death. Despite this, the anxious and non-anxious control groups failed to differ in judgments of negative valance and offensiveness, with both groups demonstrating relatively low ratings of the respective judgments. It is theoretically possible that the humorous nature of the memes leads to an attenuated perception of negative valance and offensiveness. Alternatively, consistent exposure to information regarding the current pandemic may have facilitated a form of emotional blunting in relation to Covid-19 internet memes.

Anxious individuals actively turn to social media as a means of adapting to an uncertain situation, a notion which has been evidenced in the context of the current pandemic^49^. More specifically, Cauberghe and colleagues^38^ determined that anxious individuals who use social media as a coping strategy in the context of the current pandemic, by sharing and observing humorous content related to Covid-19, were more likely to exhibit an increase in mood. Expanding these on these observations, the present study determined a disproportionate tendency for individuals presenting symptoms of anxiety to perceive Covid-19 memes as significantly more humorous, relatable and sharable. Alongside existing evidence that internet memes related to psychiatric difficulties (i.e., anxiety and depression) may be beneficial for those experiencing such difficulties^38,40^, internet memes related to the current pandemic may be constructively used to help individuals experiencing symptoms of anxiety to cope with Covid-19. Indeed, perceived social support through online interaction appears beneficial in reducing psychiatric symptoms^50^. Therefore, by sharing and observing memes related to the current pandemic, its perhaps possible that significantly anxious individuals form social and emotional bonds with others which may be perceived as socially supportive.

In the context of emotion regulation, the use of internet memes related to the current pandemic may serve to facilitate cognitive reappraisal (i.e., reinterpretation of an emotion-eliciting situation in a way that alters its meaning and changes its emotional impact)^51^. Specifically, by diminishing the meaning of a certain event, in this case the Covid-19 pandemic, while making light of a negative experience (e.g., positive reappraisal). From this perspective, anxious individuals may use memes related to the pandemic to form a humorous take on a negative experience and situation. Whilst the mediating role of cognitive reappraisal appeared to approach statistical significance (*z* = − 1.90, *p* = 0.057), no mediational effects of cognitive reappraisal or expressive suppression were evidenced. Here, the favourability of Covid-19 memes amongst anxious individuals appears to remain despite the extent of one’s ability to adequately regulate their emotions. Anxious individuals often differ in their conceptualisation of, and response to, humour when compared to heathy controls^52,53^. Indeed, relative to control subjects, individuals with social anhedonia display an impaired ability to identify humorous pictures and videos, whilst also exhibiting a more stringent inner criteria for what is deemed funny^52^. Similarly, socially anxious individuals exhibit delayed response latencies when making judgments related to humour^53^. Given the therapeutic role of humour in the symptom reduction of anxiety^54^, humour in this population may overlap with cognitive reappraisal. In other words, the use of humour itself may down-regulate negative and up-regulate positive emotions (i.e., cognitive reappraisal)^28^. Nevertheless, the role of emotion regulation warrants further investigation in the context of the current research questions.

Several limitations of the current study should be noted. The current sample consisted primarily of female student participants, and as such the present findings may not be fully generalizable to males. Moreover, whilst the present study used a comprehensive assessment to address anxious symptoms amongst the general population from the perspective of diagnostic criteria, the current outcomes cannot be extrapolated to individuals meeting diagnostic criteria for Generalized Anxiety Disorder. To that end, a replication of the current study amongst a sample meeting diagnostic criteria would be beneficial.

In sum, the perception of humour, relatability and shareability were all greater amongst individuals with symptoms of anxiety relative to non-anxious controls. Considering previous observations concerning the relationship between humour, coping, and internet memes, we tentatively suggest that memes related to the current Covid-19 pandemic may serve as beneficial coping mechanism for individuals experiencing severe symptoms of anxiety; potentially facilitating a humorous take on a negative experience and situation, and the perception of peer-support through affiliation with others. That said, further prospective work is required to confirm the causality of these claims, whereby the current objectives are replicated using a well-controlled clinical sample and the addition of control memes which are unrelated to the Covid-19 pandemic.

## Data Availability

Data will be made available on reasonable request.
