# Author Correction: Internet memes related to the COVID-19 pandemic as a potential coping mechanism for anxiety

**DOI:** 10.1038/s41598-023-36766-1

**Published:** 2023-06-21

**Authors:** Umair Akram, Kamila Irvine, Sarah F. Allen, Jodie C. Stevenson, Jason G. Ellis, Jennifer Drabble

**Affiliations:** 1 MEMElab, Department of Psychology, Sociology and Politics, Shefeld Hallam University, Collegiate Crescent, Shefeld, South Yorkshire S10 2BP UK; 2 School of Psychology, University of Lincoln, Lincoln, UK; 3 School of Social Sciences, Humanities and Law, Teesside University, Middlesbrough, UK; 4 Department of Psychology, Faculty of Health & Life Sciences, Northumbria University, Newcastle upon Tyne, UK

Correction to: *Scientifc Reports* 10.1038/s41598-021-00857-8, published online 12 November 2021

The original version of this Article contained an error, where Reference 36 was incorrectly cited. As a result, in the Introduction,

“In a survey of 133 college students, 47% of individuals reported engaging with memes as a way of alleviating psychiatric symptoms^36^.”

now reads:

“In a survey of 133 college students, 47% of individuals reported engaging with memes as a way of alleviating psychiatric symptoms^40^.”

The original Article has been corrected.

